# ATF3 is involved in rSjP40‐mediated inhibition of HSCs activation in *Schistosoma japonicum*‐infected mice

**DOI:** 10.1111/jcmm.18458

**Published:** 2024-06-21

**Authors:** Jing Li, Jiali Zhang, Bei Zhang, Guo Chen, Min Huang, Boyin Xu, Dandan Zhu, Jinling Chen, Yinong Duan, Wenxi Gao

**Affiliations:** ^1^ Department of Pathogen Biology, School of Medicine Nantong University Nantong Jiangsu People's Republic of China; ^2^ Research Center of Clinical Medicine Affiliated Hospital of Nantong University Nantong Jiangsu People's Republic of China; ^3^ Department of Laboratory Medicine People's Hospital of Haimen District Nantong Jiangsu People's Republic of China; ^4^ Department of Infection Control Affiliated Hospital of Nantong University Nantong Jiangsu People's Republic of China; ^5^ Laboratory Center, School of Educational Sciences Nantong University Nantong Jiangsu People's Republic of China

**Keywords:** ATF3, hepatic stellate cell, microRNA‐494‐3p, *Schistosoma japonicum*, *Schistosoma japonicum* protein P40

## Abstract

Schistosomiasis is a parasitic disease characterized by liver fibrosis, a process driven by the activation of hepatic stellate cells (HSCs) and subsequent collagen production. Previous studies from our laboratory have demonstrated the ability of *Schistosoma japonicum* protein P40 (SjP40) to inhibit HSCs activation and exert an antifibrotic effect. In this study, we aimed to elucidate the molecular mechanism underlying the inhibitory effect of recombinant SjP40 (rSjP40) on HSCs activation. Using a cell model in which rSjP40 inhibited LX‐2 cell activation, we performed RNA‐seq analyses and identified ATF3 as the most significantly altered gene. Further investigation revealed that rSjP40 inhibited HSCs activation partly by suppressing ATF3 activation. Knockdown of ATF3 in mouse liver significantly alleviated *S. japonicum*‐induced liver fibrosis. Moreover, our results indicate that ATF3 is a direct target of microRNA‐494‐3p, a microRNA associated with anti‐liver fibrosis effects. rSjP40 was found to downregulate ATF3 expression by upregulating microRNA‐494‐3p in LX‐2 cells. This downregulation led to the inhibition of the expression of liver fibrosis proteins α‐SMA and COL1A1, ultimately alleviating liver fibrosis caused by *S. japonicum*.

## 1 INTRODUCTION

Schistosomiasis is a parasitic disease that poses a significant threat to human health. Despite a considerable reduction in new infections due to sustained control efforts, China still has nearly 30,000 cases of advanced schistosomiasis.^1^ Liver fibrosis, a major pathological consequence of schistosomiasis, is the leading cause of mortality among patients with advanced stages of the disease.^2^ Hence, it is imperative to elucidate the molecular mechanisms underlying liver fibrosis induced by *Schistosomiasis japonicum* and identify potential molecular targets for intervention.

Remarkably, some studies have found that liver fibrosis can be ameliorated under certain conditions. During fibrosis regression, activated hepatic stellate cells (HSCs) may revert to a quiescent state or undergo apoptosis. Previous studies in our laboratory revealed a transient upregulation of α‐SMA expression in mice infected with *S. japonicum* for 12 weeks, followed by a gradual downregulation.^3^ Activation of HSCs is believed to be the central event in liver fibrosis development. Additionally, Anthony et al.^4^ reported that *S. japonicum* eggs directly inhibit HSCs activation. Previous studies demonstrated that *S. japonicum* soluble egg antigen (SEA) and its primary component, *S. japonicum* protein P40 (SjP40), modulate multiple signalling pathways to inhibit HSCs activation, thereby impeding liver fibrosis progression.^5^, ^6^ These findings imply a potential role for *S. japonicum* egg antigens in alleviating liver fibrosis. Nevertheless, the precise molecular mechanisms underlying the inhibition of liver fibrosis by SjP40 remain incompletely understood.

In this study, we employed a cell model utilizing recombinant SjP40 (rSjP40) to inhibit the activation of the human hepatic stellate cell line (LX‐2) and conducted RNA‐seq to investigate gene expression changes during this process. Our analysis identified ATF3 as the most significantly altered gene. ATF3, a member of the ATF/CREB family of transcription factors, is closely associated with cellular stress responses, including endoplasmic reticulum stress and oxidative stress. Unlike other ATF/CREB proteins, ATF3 is expressed at low levels in quiescent cells but can be rapidly induced by various cellular stresses and cytokines such as IL‐1β, TNF‐α, and TGF‐β, making it an adaptive response gene. Numerous recent studies have highlighted the pivotal role of ATF3 in fibrosis. For instance, in systemic sclerosis fibroblasts, ATF3 overexpression enhances the pro‐fibrotic effects of TGF‐β.^7^ In a mouse model of liver fibrosis induced by carbon tetrachloride, ATF3 expression in the liver is significantly upregulated and contributes to fibrosis by promoting the transcription of ACTA2 and COL1A1.^8^ However, the role of ATF3 in liver fibrosis caused by *S. japonicum* has not been investigated, and the mechanism of ATF3 in inhibiting HSCs activation by rSjP40 remain unclear.

Therefore, this study aimed to investigate the role and regulatory mechanism of ATF3 in HSCs activation and liver fibrosis using a mouse model of *S. japonicum* infection and cell function experiments.

## 2 MATERIALS AND METHODS

### 2.1 Reagents

The rSjP40 protein was obtained according to a previous protocol established in our laboratory.^9^ Antibodies against COL1A1, ATF3, and α‐SMA were sourced from Abcam (Abcam, UK), while those against GAPDH and β‐tubulin were obtained from ProteinTech Group (Chicago, USA). All secondary antibodies were purchased from Santa Cruz Biotechnology (Santa Cruz, USA).

### 2.2 Cell culture

Cells were cultured in Dulbecco's Modified Eagle's Medium supplemented with 10% heat‐inactivated foetal bovine serum and 1% penicillin/streptomycin. The human hepatic stellate cell line, LX‐2, was obtained from Nantong Third People's Hospital. The culture was maintained in a 5% CO_2_ incubator at 37°C.

### 2.3 Cell transfection

The microRNA‐NC inhibitor and microRNA‐494‐3p inhibitor were both designed by Shanghai Sangon Biotechnology Co., LTD. Lipofectamine 2000 (Thermo Fisher Scientific, Inc.) was used for the transient transfection of synthetic sequences or expression plasmids into LX‐2 cells. The cells were cultured in a corresponding medium containing 5% CO_2_ at 37°C. All cells were kept in complete culture medium for at least 24 h prior to transfection.

### 2.4 Lentivirus infection

LX‐2 cells were collected and seeded into a 24‐well plate with a cell suspension concentration of 5 × 10^4^/mL (1 mL/well). Lentivirus infection was conducted 2 h later, dividing the cells into the ATF3 overexpression group (LV‐ATF3) and the control group (LV‐NC). Uninfected cells were then removed by purinomycin screening.

### 2.5 Animal experiment

C57BL/6 mice used in this study were purchased from the Laboratory Animal Center of Nantong University and housed in a specific pathogen‐free clean room at the same facility. All animal experiments were conducted in accordance with the guidelines of the Experimental Animal Ethics Committee of Nantong University (Animal Ethics Approval number: 20180304–003).

All 6‐week‐old male C57BL/6 mice weighing approximately 20 g were obtained from the Animal Centre of Nantong University. The procedure for establishing a mouse model of *S. japonicum* infection was performed as previously described.^3^ The steps to construct the *S. japonicum* infection model in ATF3 knockdown mice were as follows: (1) Thirty C57BL/6 mice were randomly divided into two groups: AAV‐siNC (*n* = 15) and AAV‐si*Atf3* (*n* = 15). (2) Mice infected with *S. japonicum* for 2 weeks were then injected with AAV‐si*Atf3* or AAV‐siNC through the tail vein and observed for 7 weeks. (3) At the 9th week of infection, the mice were euthanized by cervical dislocation, and their liver tissues were collected. Grouping of animals for experiments has been a longstanding practice in research.

### 2.6 Western blot analysis

LX‐2 cells and liver tissues were lysed using cell lysate buffer, followed by centrifugation to obtain the supernatant. Subsequently, 20 μg of protein was electrophoresed on a 10%–12.5% SDS‐page gel and transferred to nitrocellulose. The membrane was then blocked with 5% nonfat milk and incubated with the primary antibody overnight at 4°C, followed by incubation with a secondary antibody coupled with HRP. Bands were visualized using ECL advanced reagents, and emitted light was recorded on Kodak X‐ray film using the USA Biorad, ChemiDoc XRS+.

### 2.7 RT‐qPCR analysis

Total RNA was extracted from mouse liver tissues and LX‐2 cells using the Trizol method (Takara, Japan). The concentration of mRNA was determined using an onedrop assay. The RNA was then reverse‐transcribed into cDNA using a reverse transcription kit (Thermo Fisher Scientific, USA). RT‐qPCR was performed using SYBR Premix Ex Taq™ II (Takara, Japan) on the ABI StepOnePlus system (Applied Biosystems Inc., USA). The relative mRNA expression was calculated using the 2^−ΔΔCt^ cycle method, with GAPDH serving as the internal control. Primers were provided by Shanghai Sangong Biotechnology Co., LTD. The primer sequences are listed in Table S1.

### 2.8 Wound healing assay

To assess the migration ability of LX‐2 cells, a wound‐healing assay was conducted. LX‐2 cells (1 × 10^6^ cells/well) were seeded into a 6‐well plate and incubated for 24 h at 37°C under 5% CO_2_. A wound was then created in each well by scratching the confluent monolayer with a yellow tip. After rinsing with phosphate‐buffered saline three times, cells were treated with 20 μg/mL rSjP40 for 24 h. Photos were taken immediately (*t* = 0 h), 24 h later (*t* = 24 h) or 48 h later (*t* = 48 h) under a microscope. Cell migration was assessed by measuring the size of the wound gap in at least six fields. The wound‐healing assay was performed in triplicate.

### 2.9 Migration assays

To assess the migration ability of LX‐2 cells, an analysis was conducted using a polycarbonate membrane transwell chamber containing a filter with 8 μm pores (Corning, NY, USA). LX‐2 cells were treated with 20 μg/mL rSjP40 for 24 h. Resuspended LX‐2 cells in medium without FBS were added to the upper chamber. After an 8 h incubation, cells on the polycarbonate filter were fixed with 4% paraformaldehyde and stained with 0.4% crystal violet. The cells on the top side of the filter were removed and those on the bottom side of filter were photographed under an optical microscope (200× magnification).

### 2.10 Immunohistochemistry

Paraffin sections of mouse liver tissues were incubated in an incubator at 60°C for 1 h, followed by dewaxing in xylene, hydration in gradient alcohol, antigen retrieval and treatment with 3% hydrogen peroxide. After blocking with goat serum for 30 min, sections were incubated overnight at 4°C with anti‐rabbit ATF3 monoclonal antibody (1:500, Abcam, USA), and then with a peroxidase‐labelled secondary antibody at room temperature for 30 min. Tissues were stained with diaminobenzidine (DAB) and haematoxylin, and observed under a light microscope.

### 2.11 Immunofluorescence

LX‐2 cells in the logarithmic growth stage were seeded onto cell slides in a 24‐well plate and cultured for 48 h. The culture medium was aspirated, and the cell on the slides were fixed with 4% paraformaldehyde at 4°C for 30 min, followed by three washes with PBS (5 min each) and treatment with 0.1% Triton X‐100 for 10 min. The sections were then rinsed with PBS for 5 min and blocked with 1% BSA. Subsequently, the sections were incubated with an ATF3 antibody (1:100, Abcam, USA) at 4°C overnight. After washing three times with PBS (5 min each), the slides were incubated with an anti‐rabbit secondary antibody (649 label) at room temperature for 1 h. The sections were then stained with DAPI (1:1000) for 20 min, washed three times with PBS (10 min each), and observed under an inverted fluorescence microscope.

### 2.12 Masson trichrome staining

Mouse liver sections were stained with Masson trichrome reagent to visualize collagen. The staining process involved the following steps: (1) Dewaxing and hydrating the sections; (2) Staining with Weigert's haematoxylin solution for 5–10 min; (3) Washing with water; (4) Staining with Masson's blue solution for 3–5 min followed by rinsing with water; (5) Briefly rinsing in distilled water for 1 min; (6) Staining with Ponceau acid fuchsin solution for 5–10 min; (7) Differentiating in 1% phosphomolybdic acid solution for 1–2 min; (8) Treating with 1% acetic acid for 5 min; (9) Staining with aniline blue solution for 5 min; (10) Further differentiating in 1% acetic acid for 5 min; (11) Dehydrating in multiple changes of 95% ethanol followed by anhydrous alcohol; (12) Clearing in xylene; (13) Mounting with a neutral balsam. Collagen fibres appear blue, while cytoplasm, muscle fibres and red blood cells appear red, and the nucleus appears black.

### 2.13 Target prediction

We utilized three databases, namely TargetScan (TargetScanHuman 8.0), miRDB (miRDB‐ MicroRNA Target Prediction Database) and miRtarBase (https://mirtarbase.cuhk.edu.cn/), to identify potential upstream microRNAs targeting ATF3. MicroRNA‐494‐3p was identified as having a strong complementary binding site in the 3'UTR of the *ATF3* gene.

### 2.14 Dual‐luciferase reporter assay

To directly assess the impact of microRNA‐494‐3p on ATF3, we employed the luciferase reporter method. This involved cloning the synthetic *ATF3* mutant (mut) or wild‐type (wt) 3' UTR downstream of the luciferase vector pmirGLO (Promega, WI, USA) to generate luciferase reporter plasmids *ATF3* wt and ATF3 mut. These plasmids, along with microRNA‐494‐3p mimic or mimic NC, were co‐transfected into LX‐2 cells. The Renilla luciferase expression vector pRL‐TK (TaKaRa, Dalian, China) served as an internal reference. Luciferase activity was measured using a dual luciferase assay kit (Promega, Madison, WI, USA).

### 2.15 Statistical analysis

The data are presented as mean ± SD. Normal distribution analysis was conducted using the single‐sample Kolmogorov–Smirnov nonparametric test. For statistical analysis comparing multiple groups of data, one‐way analysis of variance (ANOVA) with LSD post‐hoc test was employed. The independent sample *T*‐test was used to analyse the comparison between two groups of data. A significance level of *p* < 0.05 was considered statistically significant. Data visualization was performed using GraphPad Prism 5.0 statistical software.

## 3 RESULTS

### 3.1 ATF3 down‐regulation in chronic *S. japonicum* Infection

**FIGURE 1:**
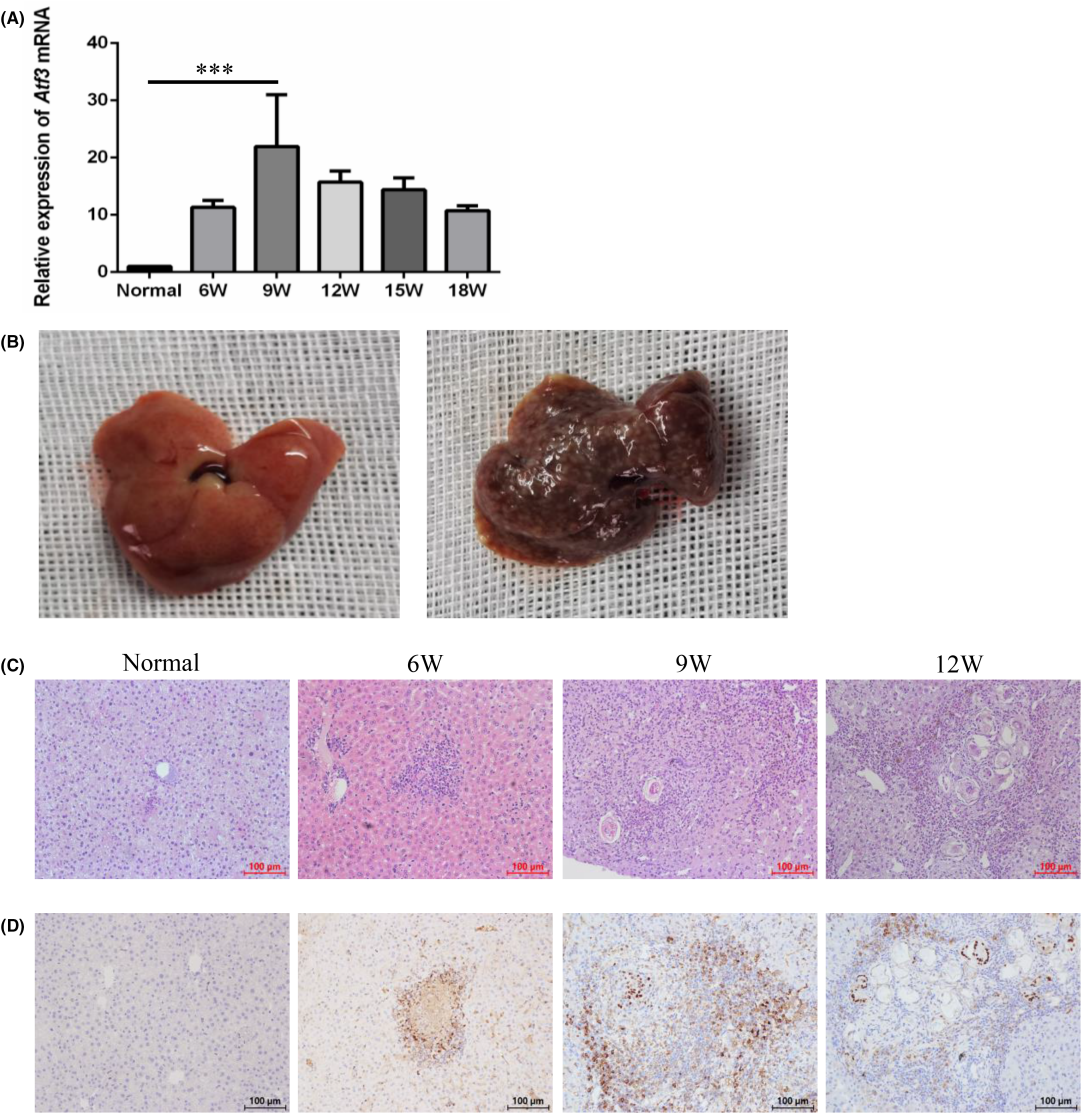
*Atf3* mRNA expression in the liver of *S. japonicum*‐infected mice. (A) *Atf3* mRNA expression at different weeks post *S. japonicum* infection, as detected by RT‐qPCR. (B) Liver tissue from an *S. japonicum*‐infected mouse model. (C) HE staining of liver tissue from *S. japonicum*‐infected mice at different weeks. (D) Immunohistochemical staining of liver tissue from *S. japonicum*‐infected mice at different weeks. ***p < 0.001 compared with the normal group.

We conducted RNA‐seq analysis to investigate gene expression changes in LX‐2 cells following treatment with rSjP40. Differential gene expression analysis revealed ATF3 as the most significantly altered gene. Therefore, we focused on elucidating the role of ATF3 in liver fibrosis induced by *S. japonicum*. First, we established a mouse model of *S. japonicum* infection. Post‐infection, the liver surface exhibited a brown hue and was covered with grey and white nodules of eggs, with a noticeable increase in texture hardness (Figure 1B). Histological examination using haematoxylin and eosin (HE) staining further confirmed the successful establishment of the model. Over time, liver tissue granulomas progressively increased, correlating with a deterioration in liver fibrosis (Figure 1C). RT‐qPCR analysis revealed a gradual increase in *Atf3* mRNA expression at the 6th week post‐infection, peaking at the 9th week (*p* <0.001). However, *Atf3* mRNA expression decreased by the 12th week post‐infection, with this declining trend persisting until the 18th week (Figure 1A). Immunohistochemical staining results were consistent with the RT‐qPCR results (Figure 1D).

### 3.2 rSjP40 inhibits LX‐2 cell activation by inhibiting ATF3

**FIGURE 2:**
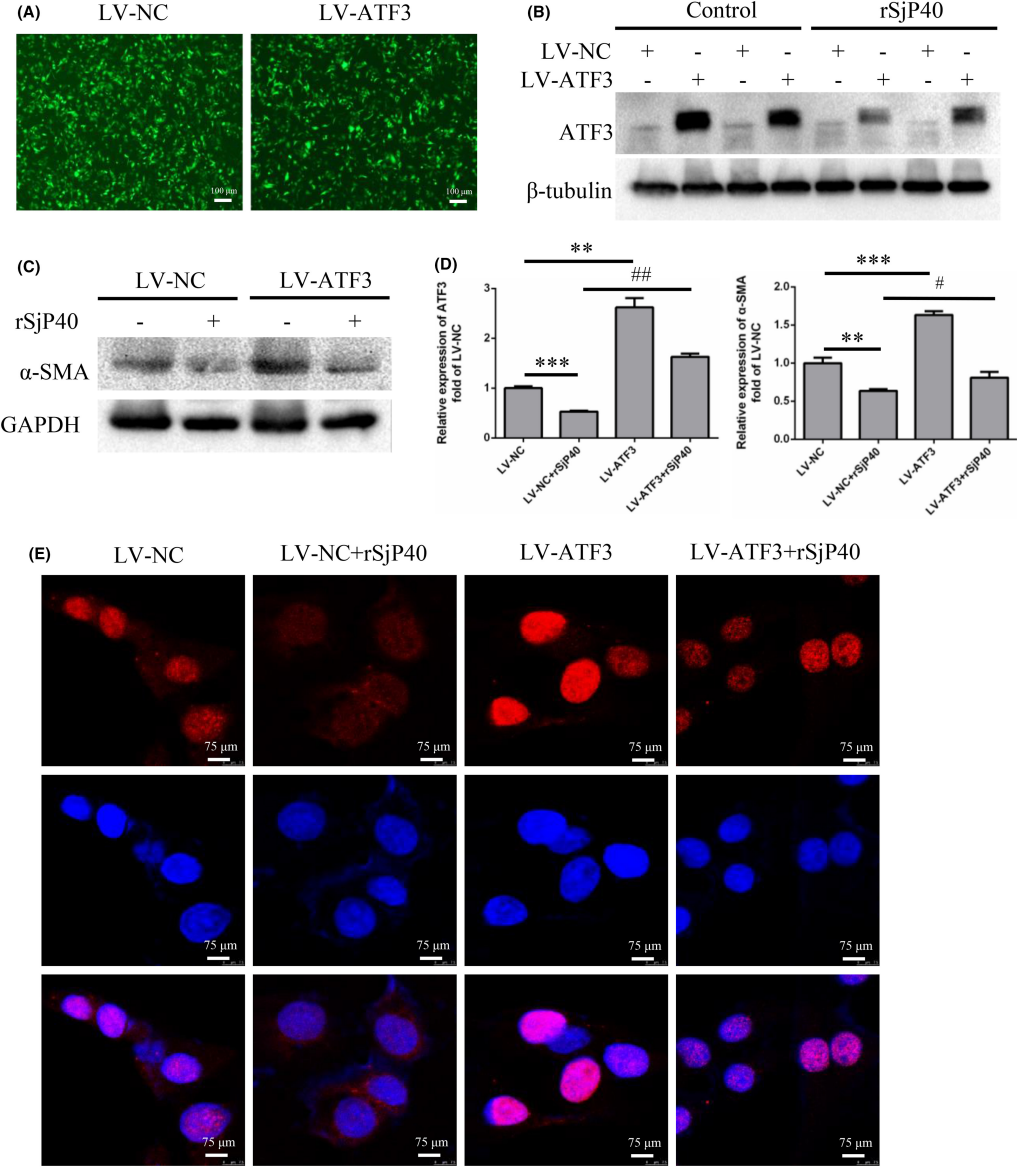
rSjP40 inhibits α‐SMA expression in LX‐2 cell by down‐regulating ATF3. (A) Infection of LX‐2 cells with LV‐ATF3 lentivirus, observed for GFP fluorescence intensity. (B) Detection of ATF3 overexpression efficiency and the effect of rSjP40 treatment on ATF3 protein expression by Western blot. (C) Detection of the effect of rSjP40 on α‐SMA expression in LX‐2 cells stably overexpressing ATF3 by Western blot. (D) Quantitative statistical analysis of Western blot results. (E) Immunofluorescence showing ATF3 expression in the nucleus after rSjP40 treatment. (Red: ATF3 staining; Blue: DAPI staining for nucleus). **p < 0.01 compared to LV‐NC group, ***p < 0.001 compared to LV‐NC group, #*p* < 0.05 compared to LV‐NC + rSjP40 group, ^##^ *p* < 0.01 compared to LV‐NC + rSjP40 group.

To further elucidate the role of ATF3 in rSjP40‐mediated inhibition of LX‐2 cell activation, we established ATF3‐overexpressing LX‐2 cells (Figure 2A,B). Western blot analysis revealed that while rSjP40 effectively suppressed α‐SMA expression, this inhibition was notably reversed upon ATF3 overexpression (Figure 2C,D). Moreover, we observed that ATF3 overexpression alone significantly promoted α‐SMA expression (Figure 2C,D). Previous studies have shown that in a mouse model of liver fibrosis induced by CCl_4_, ATF3 expression in the nucleus of HSCs is increased, leading to the upregulation of pro‐fibrotic factors such as α‐SMA and COL1A1.^8^ In our study, we also investigated changes in nuclear ATF3 expression following rSjP40 treatment, revealing a significant reduction in nuclear ATF3 expression post‐treatment (Figure 2E). Thus, we hypothesized that the decrease in α‐SMA expression following rSjP40 treatment might be attributed to reduced nuclear ATF3 expression.

**FIGURE 3:**
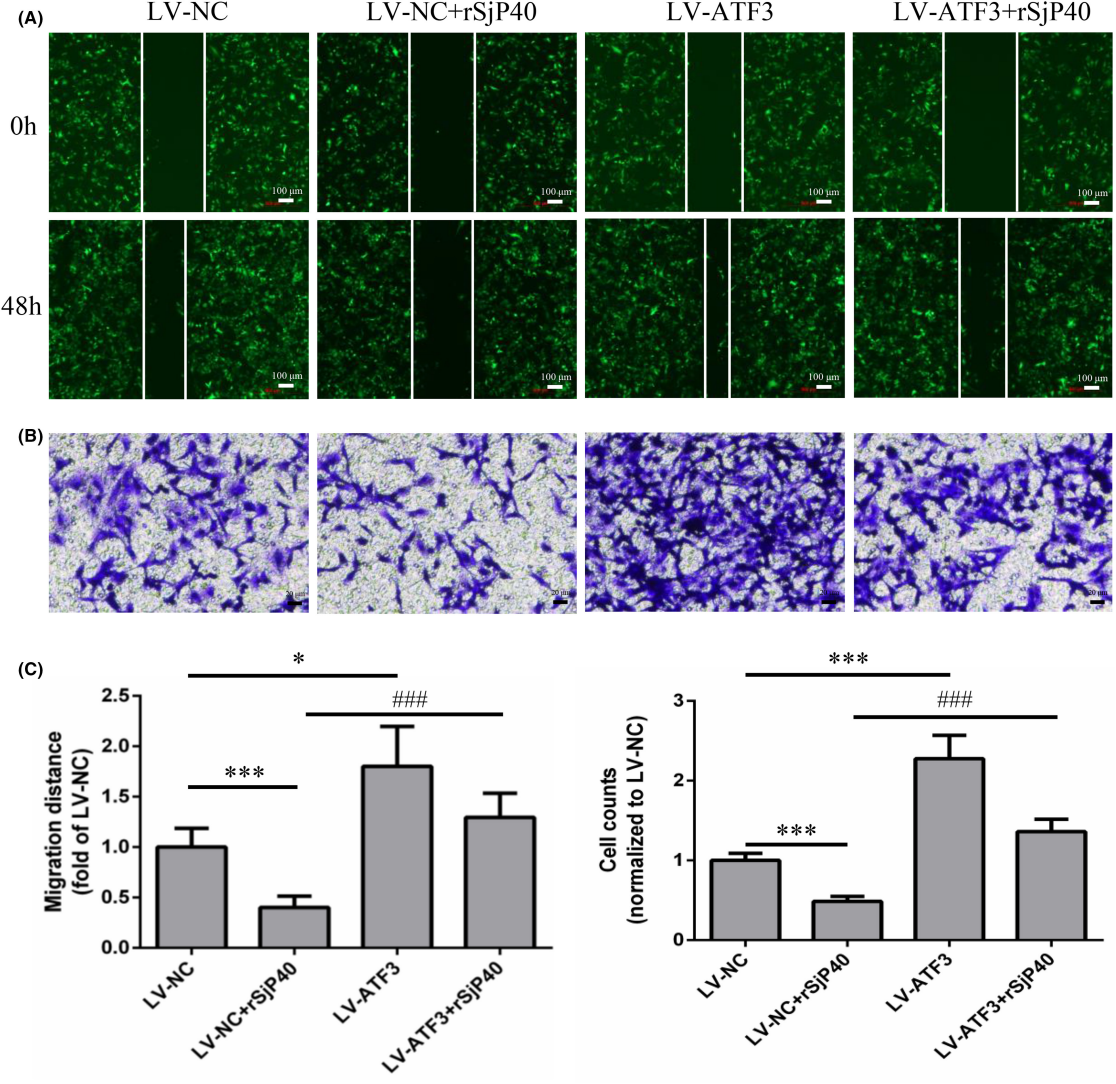
Effect of ATF3 overexpression on rSjP40 inhibition of LX‐2 cell migration. (A) Cell scratch assay after LX‐2 cells were infected with LV‐ATF3 and LV‐NC Lentivirus (green fluorescence represents virus‐infected positive cells). (B) Transwell assay after LX‐2 cells were infected with LV‐ATF3 and LV‐NC lentivirus. (C) Quantitative statistical analysis of cell scratch and transwell assays. *p < 0.05 compared to LV‐NC group; ***p < 0.001 compared to LV‐NC group; ^###^ *p* < 0.001 compared to LV‐NC + rSjP40 group.

Cell migration is crucial for tissue damage repair and inflammatory responses, and the migration of activated HSCs plays a key role in liver fibrosis progression. Therefore, we further explored the impact of ATF3 on rSjP40‐mediated inhibition of LX‐2 cell activation using wound healing and transwell assays. Our results demonstrated that rSjP40 significantly suppressed LX‐2 cell migration, whereas ATF3 overexpression substantially promoted this migration (Figure 3A–C). These results suggest that ATF3 is essential for rSjP40's inhibitory effect on LX‐2 cell activation.

### 3.3 ATF3 knockdown alleviates *S. japonicum* induced liver fibrosis

**FIGURE 4:**
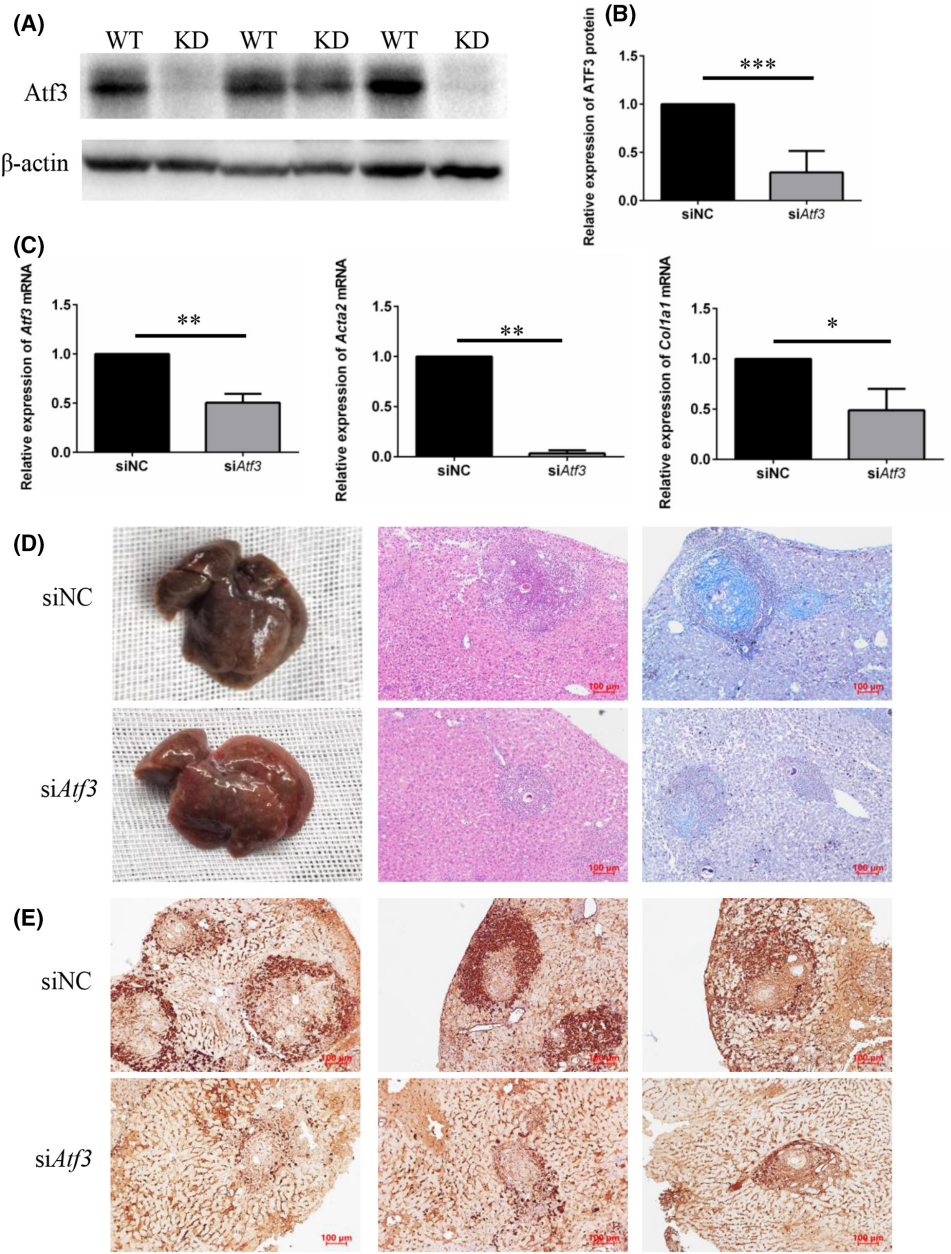
Reducing ATF3 expression ameliorates *S. japonicum*‐induced liver fibrosis. (A) Detection of ATF3 protein expression in mouse liver tissues by Western blot (WT: wild‐type mice, KD: ATF3 knockdown mice). (B) Quantitative statistical analysis of ATF3 knockdown efficiency in mouse liver tissue by Western blot. (C) Detection of *Atf3*, *Acta2* and *Col1a1* mRNA expression in mouse liver tissue by RT‐qPCR. (D) Evaluation of liver fibrosis by macroscopic examination, H&E, and Masson staining. (E) Immunohistochemical staining for ATF3, α‐SMA, and COL1A1 (scale: 100 μm). **p < 0.01 compared to shNC group.

To investigate the role of ATF3 in liver fibrogenesis in vivo, we established a *S. japonicum* infection model in ATF3 knockdown mice. We first assessed the efficiency of ATF3 knockdown in mice using western blot and RT‐qPCR. Compared to the siNC group, the si*Atf3* group exhibited significantly decreased ATF3 expression in liver tissue (Figure 4A–C). RT‐qPCR results also revealed significantly reduced mRNA expression levels of *Acta2* and *Col1a1* in liver tissues of the si*Atf3* group compared to the siNC group (Figure 4C).

Furthermore, livers from the siNC group displayed typical liver fibrosis characteristics, appearing dark brown in colour with a hard, dense texture and numerous grey and white egg nodules on the surface. In contrast, livers from the si*Atf3* group exhibited reduced liver fibrosis, with many grey and white egg nodules still present but with a reddish‐brown colour and relatively soft texture (Figure 4D). HE and Masson staining of liver tissue sections revealed a significant reduction in the extent of liver granulomas and blue collagen in the si*Atf3* group compared to the siNC group (Figure 4D). These results collectively indicate that ATF3 knockdown significantly mitigates liver fibrosis induced by *S. japonicum*.

Immunohistochemical staining was used to assess the expression of fibrosis markers in liver tissues after ATF3 knockdown. The results demonstrated a significant downregulation of α‐SMA and COL1A1 protein levels in liver tissues of the si*Atf3* group compared to the siNC group (Figure 4E). Taken together, these results indicate that ATF3 knockdown in liver tissue of mice significantly alleviates liver fibrosis induced by *S. japonicum*.

### 3.4 TLR‐4 contributes to ATF3 down‐regulation induced by rSjP40

**FIGURE 5:**
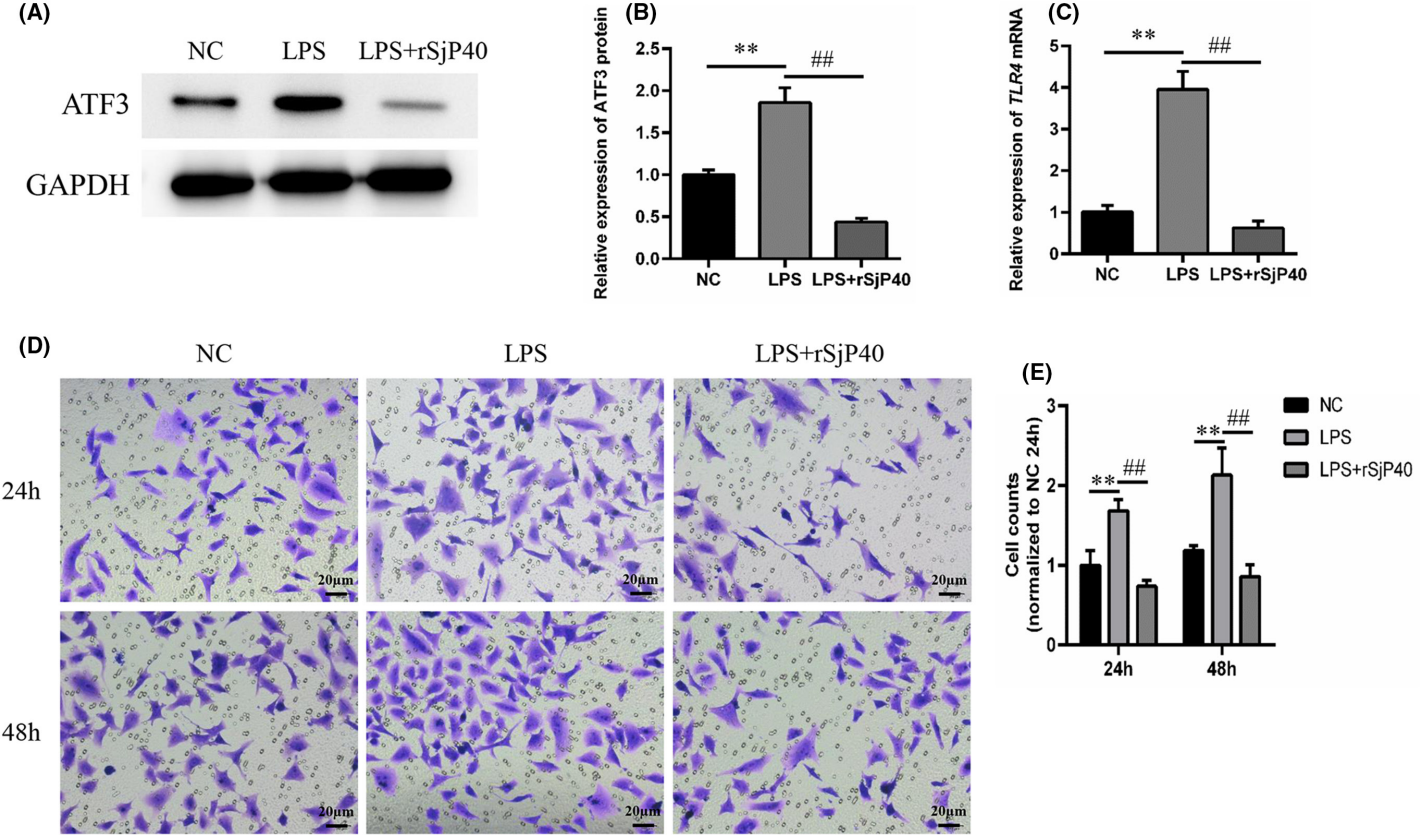
TLR‐4 contributes to down‐regulation of ATF3 expression induced by rSjP40. (A) Detection of ATF3 expression in control group, LPS group, or LPS + rSjP40 group by Western blot. (B) Quantitative statistical analysis of ATF3 expression in each group by Western blot. (C) Detection of TLR4 expression in control group, LPS group, or LPS + rSjP40 group by RT‐qPCR. (D) Transwell assay to determine the migration ability of LX‐2 cells in control group, LPS group, or LPS + rSjP40 group. (E) Quantitative statistical analysis of transwell assays. **p < 0.01 compared to NC group; ^##^ *p* < 0.01 compared to LPS group.

Previous laboratory studies have demonstrated that rSjP40 induces HSCs senescence by inhibiting the cell membrane surface receptor TLR4. This led us to speculate whether TLR4 signalling is involved in the inhibition of ATF3 expression and LX‐2 cell activation by rSjP40. RT‐qPCR analysis revealed that rSjP40 suppressed the expression of TLR4 mRNA induced by LPS (Figure 5C). Additionally, we observed that while LPS treatment upregulated ATF3 expression in LX‐2 cells, this upregulation was significantly inhibited in the LPS + rSjP40 group (Figure 5A,B). Transwell assay results showed that LPS treatment enhanced the migration ability of LX‐2 cells, which was then significantly inhibited by rSjP40 treatment (Figure 5D,E). These results suggest that rSjP40 may inhibit ATF3 expression and LX‐2 cell activation by suppressing TLR4 signalling.

### 3.5 rSjP40 Inhibits ATF3 expression in LX‐2 cell by up‐regulating MicroRNA‐494‐3p

**FIGURE 6:**
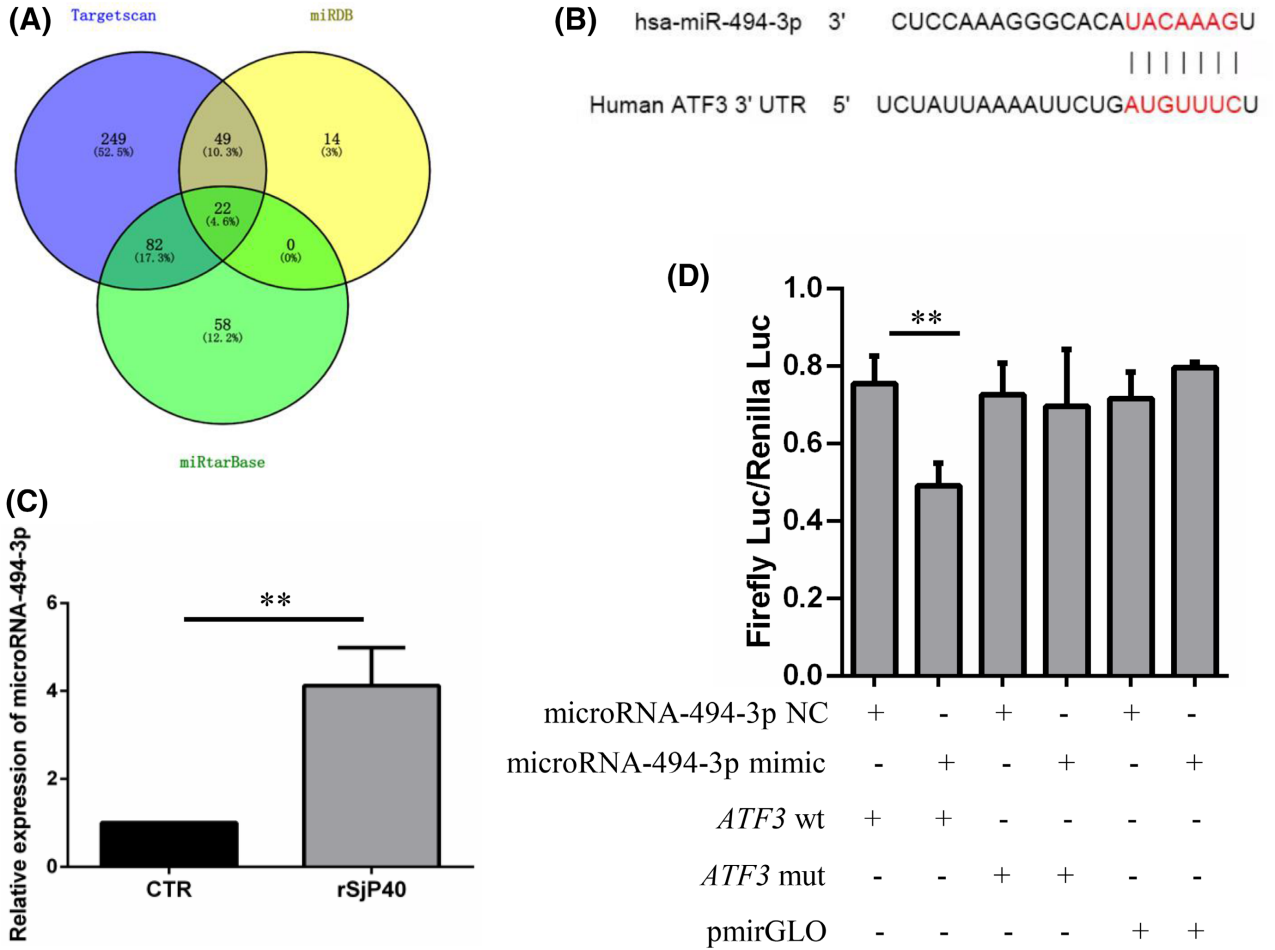
MicroRNA‐494‐3p targets ATF3. (A) Prediction of upstream microRNAs of ATF3 using three databases (Targetscan, miRDB, and miRtarBase). (B) Potential binding site between microRNA‐494‐3p and ATF3. (C) Detection of the effect of rSjP40 on microRNA‐494‐3p expression in LX‐2 cells by RT‐qPCR. (D) Detection of the combination of microRNA‐494‐3p and ATF3 3’ UTR by double luciferase reporter assay. **p < 0.01 compared to NC group.

There is growing evidence that host microRNAs are dysregulated following *S. japonicum* infection, and aberrant microRNA expression is a pivotal event in the progression of liver fibrosis, directly influencing pro‐fibrosis and anti‐fibrosis pathways. To elucidate the molecular mechanism underlying rSjP40‐mediated inhibition of ATF3 in alleviating liver fibrosis, we predicted the upstream microRNAs targeting ATF3 using multiple databases, including TargetScan, miRDB and miRtarBase. These databases collectively identified 402, 85 and 162 microRNAs potentially targeting ATF3, respectively. Among the shared predictions, microRNA‐494‐3p exhibited the highest score and conservation across species (Figure 6A). Figure 6B illustrated the potential binding sites between microRNA‐494‐3p and the ATF3 3’ UTR region, which are highly conserved between human and mouse species. Furthermore, the expression of microRNA‐494‐3p was significantly upregulated following rSjP40 treatment, contrasting with ATF3 expression (Figure 6C). Subsequently, we employed a dual luciferase reporter assay to confirm the direct binding of microRNA‐494‐3p to ATF3 in LX‐2 cells. The results demonstrated that overexpression of microRNA‐494‐3p reduced the luciferase intensity of the wild‐type *ATF3* 3’ UTR reporter plasmid, while having no effect on the luciferase activity of the mutant‐type *ATF3* 3’ UTR reporter plasmid (Figure 6D), indicating a direct interaction between microRNA‐494‐3p and ATF3.

**FIGURE 7:**
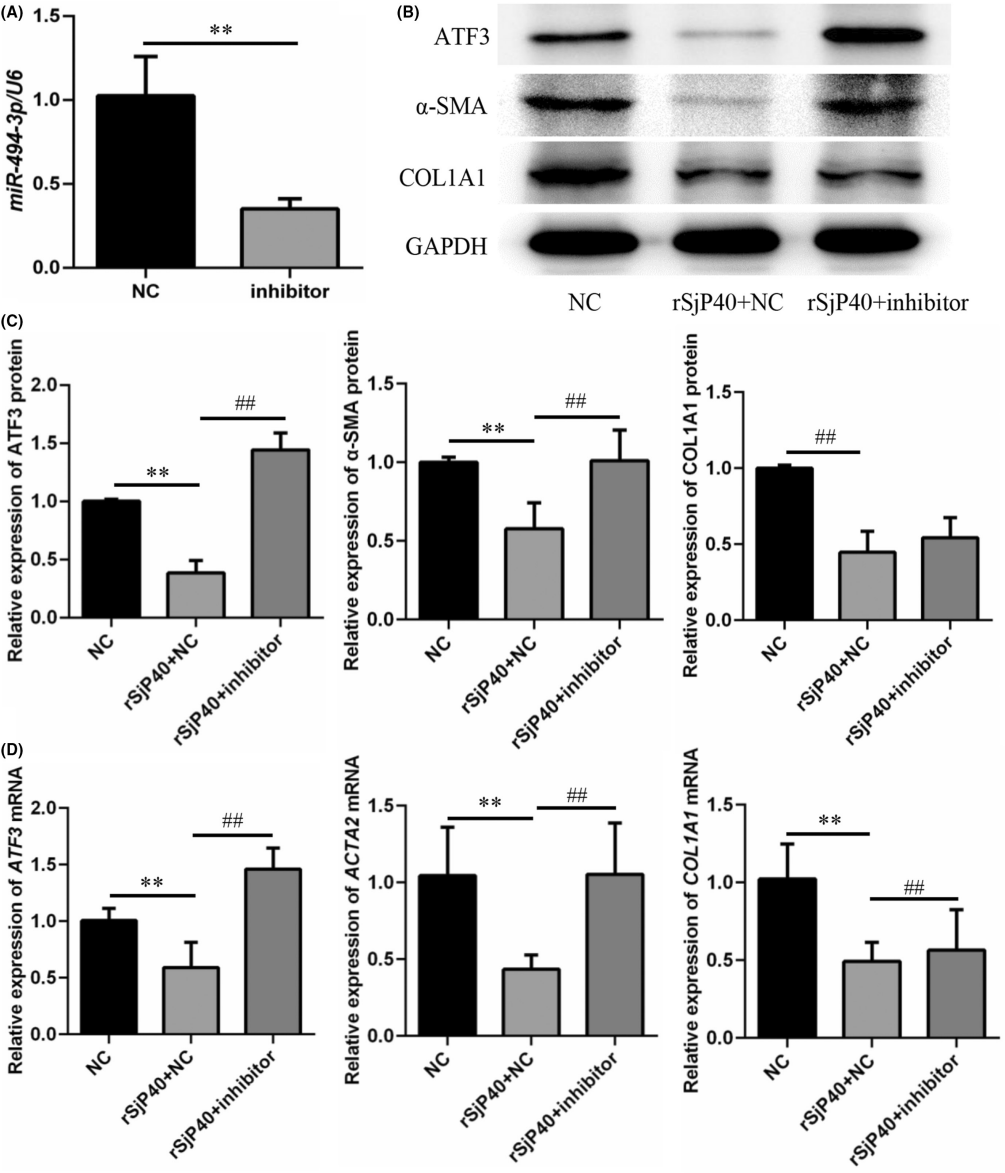
MicroRNA‐494‐3p negatively regulates ATF3, α‐SMA and COL1A1 expression. (A) Detection of inhibitory efficiency of microRNA‐494‐3p inhibitor by RT‐qPCR. (B, C) Changes in ATF3, α‐SMA, and COL1A1 protein levels upon silenced expression of microRNA‐494‐3p. (D) Changes in *ATF3*, *ACTA2*, and *COL1A1* mRNA levels upon silenced expression of microRNA‐494‐3p. **p < 0.01 compared to NC group; ^##^ *p* < 0.01 compared to rSjP40 + NC group.

To further validate the impact of microRNA‐494‐3p on rSjP40‐mediated inhibition of ATF3, a microRNA‐494‐3p inhibitor was synthesized for subsequent experiments. LX‐2 cells were transfected with either a microRNA‐NC inhibitor or a microRNA‐494‐3p inhibitor, and their inhibitory efficiencies were assessed using RT‐qPCR. The microRNA‐494‐3p inhibitor group exhibited a 65.9% inhibition efficiency compared to the microRNA‐NC inhibitor group (Figure 7A). Western blot and RT‐qPCR analyses revealed that, compared to the rSjP40 + NC group, the rSjP40 + inhibitor group showed significantly upregulated expression of ATF3 and α‐SMA (Figure 7B–D). Although the microRNA‐494‐3p inhibitor transfection did not significantly alter COL1A1 protein expression, it showed an increasing trend (Figure 7B,C). These results indicate that the microRNA‐494‐3p inhibitor effectively reverses the inhibitory effect of rSjP40 on ATF3 and fibrosis marker expression.

**FIGURE 8:**
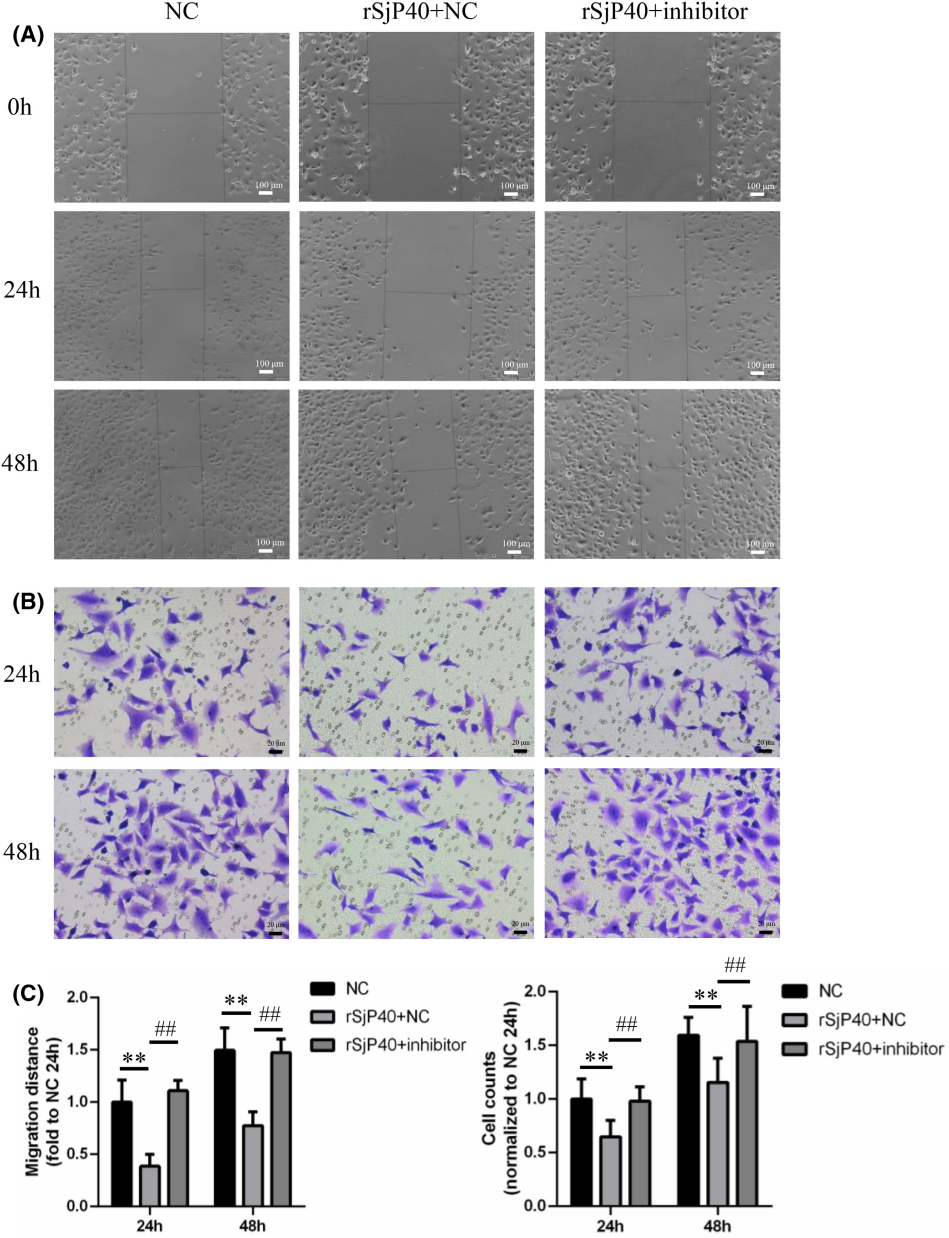
Effects of microRNA‐494‐3p inhibitor on rSjP40 inhibition of LX‐2 cell migration. (A) Impact of microRNA‐494‐3p inhibitor on LX‐2 cell migration assayed by cell scratch assay. (B) Impact of microRNA‐494‐3p inhibitor on LX‐2 cell migration assayed by transwell assay. (C) Quantitative statistical analysis of cell scratch and transwell assays. **p < 0.01 compared to NC group; ^##^ *p* < 0.01 compared to rSjP40 + NC group.

Furthermore, we investigated the impact of microRNA‐494‐3p inhibitors on rSjP40‐mediated inhibition of LX‐2 cell migration. The results revealed that the microRNA‐494‐3p inhibitor group exhibited significantly reduced LX‐2 cell migration compared to the NC inhibitor group following rSjP40 treatment (Figure 8). These results suggest that microRNA‐494‐3p exerts an anti‐fibrotic effect in liver fibrosis, which contradicts the biological function of ATF3.

## 4 DISCUSSION

In our previous study, we demonstrated that rSjP40 inhibits HSCs activation through various mechanisms. Specifically, rSjP40 was shown to suppress HSCs proliferation and activation by upregulating microRNA‐155, which targets FOXO3a for degradation.^10^ Additionally, rSjP40 was found to reduce HSCs activation and COL1A1 synthesis by increasing Let‐7b expression.^11^ Moreover, rSjP40 induces senescence in activated HSCs via the STAT3/p53/p21 and SKP2/p27 signalling pathways, thus inhibiting HSCs activation.^9^, ^12^ Furthermore, rSjP40 inhibits HSCs activation by promoting the nuclear translocation of YB1 and inducing the BMP‐7/Smad1/5/8 pathway.^13^

In the current study, we utilized RNA‐seq to investigate gene expression changes during rSjP40‐mediated inhibition of LX‐2 cell activation. Sequencing analysis revealed a substantial alteration in gene expression upon rSjP40 treatment, with ATF3 exhibiting the most significant downregulation. ATF3 has been implicated in fibrosis, with studies showing its upregulation in the CCL4‐induced mouse liver fibrosis model.^8^ However, the role of ATF3 in *S. japonicum*‐induced liver fibrosis remains unknown. Our animal experiments revealed a gradual increase in ATF3 mRNA expression at 6 weeks post‐infection, peaking at 9 weeks, and decreasing thereafter, similar to the expression trend of α‐SMA. In vitro experiments demonstrated that ATF3 overexpression significantly reversed α‐SMA expression and LX‐2 cell migration inhibited by rSjP40. ATF3 is known to translocate to the nucleus upon activation, where it regulates the transcription of target genes.^14^ Therefore, we investigated whether the cellular localization of ATF3 in LX‐2 cells changed following rSjP40 treatment using confocal microscopy. The results showed a significant inhibition of ATF3 distribution in the nucleus. Additionally, overexpressed ATF3 was predominantly located in the nucleus and could reverse the expression of α‐SMA inhibited by rSjP40. These results suggest that ATF3 may be involved in rSjP40‐mediated inhibition of LX‐2 activation by promoting the transcription of the target gene α‐SMA through binding to its promoter.

TLR, especially the TLR4 signalling pathway, plays a crucial role in liver fibrosis.^15^ LPS, a ligand of TLR4, is closely associated with HSCs activation. Previous studies have demonstrated that rSjP40 induces senescence in LX‐2 cells by inhibiting the TLR‐4 pathway. In our study, we found that TLR4 signalling is involved in the inhibition of ATF3 expression and LX‐2 cell activation induced by rSjP40.

There is increasing evidence suggesting that host microRNAs are dysregulated following *S. japonicum* infection.^16^ Dysregulation of microRNA expression is a key event in liver fibrosis progression and directly affects pro‐fibrosis and anti‐fibrosis pathways.^17^, ^18^ MicroRNA‐155 has been reported to be involved in rSjP40 inhibition of HSCs activation by targeting FOXO3a expression directly.^10^ Additionally, rSjP40 inhibits LX‐2 cell activation by upregulating PPARγ expression through microRNA‐27b.^19^ These studies indicate that rSjP40 can influence the expression of key fibrosis‐related genes by regulating multiple microRNAs. Therefore, we investigated whether rSjP40 inhibits ATF3 expression by regulating microRNA expression levels. Using microRNA prediction databases, we identified microRNA‐494‐3p, a highly conserved microRNA with the highest score, as a potential regulator of ATF3. Further analysis revealed that microRNA‐494‐3p expression in LX‐2 cells was significantly upregulated following rSjP40 treatment. A double luciferase reporter assay confirmed that microRNA‐494‐3p directly binds to ATF3. Moreover, transfection of microRNA‐494‐3p inhibitor into LX‐2 cells significantly reversed the antifibrotic effect of rSjP40.

In conclusion, this study provides a comprehensive analysis of the microRNA‐494‐3p/ATF3/α‐SMA signalling axis in rSjP40‐mediated inhibition of LX‐2 cell activation, highlighting ATF3 as a potential therapeutic target for liver fibrosis.

## AUTHOR CONTRIBUTIONS

**Jing Li:** Data curation (lead); formal analysis (equal); methodology (equal); writing – original draft (lead). **Jiali Zhang:** Formal analysis (equal); investigation (equal); methodology (equal). **Bei Zhang:** Formal analysis (equal); methodology (equal). **Guo Chen:** Methodology (equal). **Min Huang:** Methodology (supporting). **Boyin Xu:** Resources (supporting). **Dandan Zhu:** Resources (supporting); writing – review and editing (supporting). **Jinling Chen:** Resources (supporting). **Yinong Duan:** Conceptualization (equal); project administration (lead); resources (equal); supervision (equal); writing – review and editing (equal). **Wenxi Gao:** Conceptualization (equal); investigation (equal); resources (equal); software (lead); supervision (equal).

## FUNDING INFORMATION

This work was supported by National Natural Science Foundation of China (Grant Numbers: 82172295, 81871677).

## CONFLICT OF INTEREST STATEMENT

The authors confirm that there are no conflicts of interest.

## CONSENT

Not applicable.

## Supporting information


Table S1.

## Data Availability

The data presented in this study are available on request from the corresponding author.
