# Supplementary material for: ATF3 is involved in rSjP40‐mediated inhibition of HSCs activation in Schistosoma japonicum‐infected mice
Source: J Cell Mol Med. 2024 Jun 21;28(12):e18458. doi: 10.1111/jcmm.18458 (PMC11190947; doi:10.1111/jcmm.18458)
Supplement: Supplementary file 1 — Table S1. [file JCMM-28-e18458-s001.docx]

| Table S1 List of all primers in RT-qPCR. | | |
| --- | --- | --- |
| Primer name | Sequence（5’-3’） | Base number |
| h*GAPDH*-F | GGAGCGAGATCCCTCCAAAAT | 21 |
| h*GAPDH*-R | GGCTGTTGTCATACTTCTCATGG | 23 |
| h*ATF3*-F | CCTCTGCGCTGGAATCAGTC | 20 |
| h*ATF3*-R | TTCTTTCTCGTCGCCTCTTTT | 21 |
| h*COL1A1*-F | GGGACACAGAGGTTTCAGT | 19 |
| h*COL1A1*-R | CACCATCATTTCCACGAGC | 19 |
| h*ACTA2*-F | ACGAAGCACAGAGCAAAAG | 19 |
| h*ACTA2*-R | GCCTGGATAGCCACATACA | 19 |
| h*TLR4*-F | GGGTATTTGACACCCTCCATAG | 22 |
| h*TLR4*-R | CAAGAGTGCTGAGGGAATACAG | 22 |
| m*Gapdh*-F | TGGAAAGCTGTGGCGTGAT | 19 |
| m*Gapdh*-R | TGCTTCACCACCTTCTTGAT | 20 |
| m*Atf3*-F | GAGGATTTTGCTAACCTGACACC | 23 |
| m*Atf3*-R | TTGACGGTAACTGACTCCAGC | 21 |
| m*Col1a1*-F | CACTCCTATCTCTCTCCTGTTGCCT | 25 |
| m*Col1a1*-R | CACATCTTGTTCCTGTGTGCTCTCT | 25 |
| m*Acta2*-F | CACTCCTATCTCTCTCCTGTTGCCT | 25 |
| m*Acta2*-R | CACATCTTGTTCCTGTGTGCTCTCT | 25 |
| hsa-microRNA-494-3p-F | ACACTCCAGCTGGGTGAAACATACACGGGA |  |
| hsa-microRNA-494-3p-R | CTCAACTGGTGTCGTGGAGTCGGCAATTCAGTTGAGGAGGTTTC |  |
| U6-F | GAGGCGACGAGAAAGAAA |  |
| U6-R | GCCGATGAAGGTTGAGCA |  |
| URP | GGGACACAGAGGTTTCAGT |  |
